# Blockade of growth hormone receptor signaling by using pegvisomant: A functional therapeutic strategy in hepatocellular carcinoma

**DOI:** 10.3389/fonc.2022.986305

**Published:** 2022-10-06

**Authors:** Ahmed O. Kaseb, Abedul Haque, Deeksha Vishwamitra, Manal M. Hassan, Lianchun Xiao, Bhawana George, Vishal Sahu, Yehia I. Mohamed, Roberto Carmagnani Pestana, Jamie Lynne Lombardo, Rony Avritscher, James C. Yao, Robert A. Wolff, Asif Rashid, Jeffrey S. Morris, Hesham M. Amin

**Affiliations:** ^1^ Department of Gastrointestinal Medical Oncology, The University of Texas MD Anderson Cancer Center, Houston, TX, United States; ^2^ Department of Hematopathology, The University of Texas MD Anderson Cancer Center, Houston, TX, United States; ^3^ Department of Epidemiology, The University of Texas MD Anderson Cancer Center, Houston, TX, United States; ^4^ Department of Biostatistics, The University of Texas MD Anderson Cancer Center, Houston, TX, United States; ^5^ Department of Pathology, The University of Texas MD Anderson Cancer Center, Houston, TX, United States; ^6^ Department of Interventional Radiology, The University of Texas MD Anderson Cancer Center, Houston, TX, United States; ^7^ MD Anderson Cancer Center UTHealth Houston Graduate School of Biomedical Sciences, Houston, TX, United States

**Keywords:** hepatocellular carcinoma, growth hormone receptor, pegvisomant, targeted therapy, somavert

## Abstract

Hepatocellular carcinoma (HCC) is an aggressive neoplasm with poor clinical outcome because most patients present at an advanced stage, at which point curative surgical options, such as tumor excision or liver transplantation, are not feasible. Therefore, the majority of HCC patients require systemic therapy. Nonetheless, the currently approved systemic therapies have limited effects, particularly in patients with advanced and resistant disease. Hence, there is a critical need to identify new molecular targets and effective systemic therapies to improve HCC outcome. The liver is a major target of the growth hormone receptor (GHR) signaling, and accumulating evidence suggests that GHR signaling plays an important role in HCC pathogenesis. We tested the hypothesis that GHR could represent a potential therapeutic target in this aggressive neoplasm. We measured GH levels in 767 HCC patients and 200 healthy controls, and then carried out clinicopathological correlation analyses. Moreover, specific inhibition of GHR was performed *in vitro* using siRNA and pegvisomant (a small peptide that blocks GHR signaling and is currently approved by the FDA to treat acromegaly) and *in vivo*, also using pegvisomant. GH was significantly elevated in 49.5% of HCC patients, and these patients had a more aggressive disease and poorer clinical outcome (P<0.0001). Blockade of GHR signaling with siRNA or pegvisomant induced substantial inhibitory cellular effects *in vitro.* In addition, pegvisomant potentiated the effects of sorafenib (P<0.01) and overcame sorafenib resistance (P<0.0001) *in vivo.* Mechanistically, pegvisomant decreased the phosphorylation of GHR downstream survival proteins including JAK2, STAT3, STAT5, IRS-1, AKT, ERK, and IGF-IR. In two patients with advanced-stage HCC and high GH who developed sorafenib resistance, pegvisomant caused tumor stability. Our data show that GHR signaling represents a novel “druggable” target, and pegvisomant may function as an effective systemic therapy in HCC. Our findings could also lead to testing GHR inhibition in other aggressive cancers.

## Introduction

Hepatocellular carcinoma (HCC) is an aggressive neoplasm associated with poor clinical outcome and, therefore, is considered a major health problem worldwide (1). Since the early 1980s, the incidence of HCC has tripled in the US, and it is currently the fastest growing cause of cancer-related mortality. This is because up to 80% of HCC patients are diagnosed at an advanced stage with underlying cirrhosis and/or fibrosis, which precludes curative treatment options such as surgical resection and liver transplantation (2).

Systemic treatment options for HCC are limited. Sorafenib, an orally available tyrosine kinase inhibitor (TKI), demonstrated a modest but significant overall survival (OS) benefit as compared to placebo (10.7 vs. 7.9 months, hazard ratio (HR) 0.69; P<0.001) (3). Additional TKI including lenvatinib, regorafenib, ramucirumab, and cabozantinib also demonstrated slight OS improvements ranging from 1.6 to 2.8 months vs. placebo (4–7). Immune checkpoint blockade was also evaluated in HCC. Phase I/II studies showed modest effects of the anti-PD-1 antibodies nivolumab and pembrolizumab as single therapies in HCC patients who progressed on sorafenib (8, 9). Most recently, the FDA approved the combination of atezolizumab (anti-PD-L1) plus bevacizumab (anti-VEGF) as first-line therapy, based on phase III IMbrave150 trial, which assessed this combination vs. sorafenib and yielded longer progression-free survival (PFS) of 6.8 vs. 4.3 months, HR 0.59; P<0.001, and objective response rate of 27% vs. 12% (10). However, more recent studies suggest that immune checkpoint blockade may not be effective in HCC when associated with non-alcoholic steatohepatitis and hepatic fibrosis (11–13). Therefore, progress is needed and effective systemic therapies against HCC remain an unmet need.

The growth hormone (GH) receptor (GHR) is a prototype class I cytokine receptor, and the liver is considered a key organ in GHR signaling (14). Recently, the contribution of GHR signaling to the development, survival, and progression of cancer has gained increasing attention (15). Notably, patients with Laron syndrome, who have *GHR* gene deficiency, rarely develop cancer (16). Conversely, in a population-based study, patients treated with human pituitary GH were found to have a significantly higher risk of dying from cancer (17). It is believed that in cancer cells, binding of GH with GHR activates receptor associated intracellular domain such as JAK2, which further accelerate activation of oncogenic transcription factors STAT3 and STAT5, and other survival molecules including IRS-1, AKT, and ERK (18–20).

**Figure 1:**
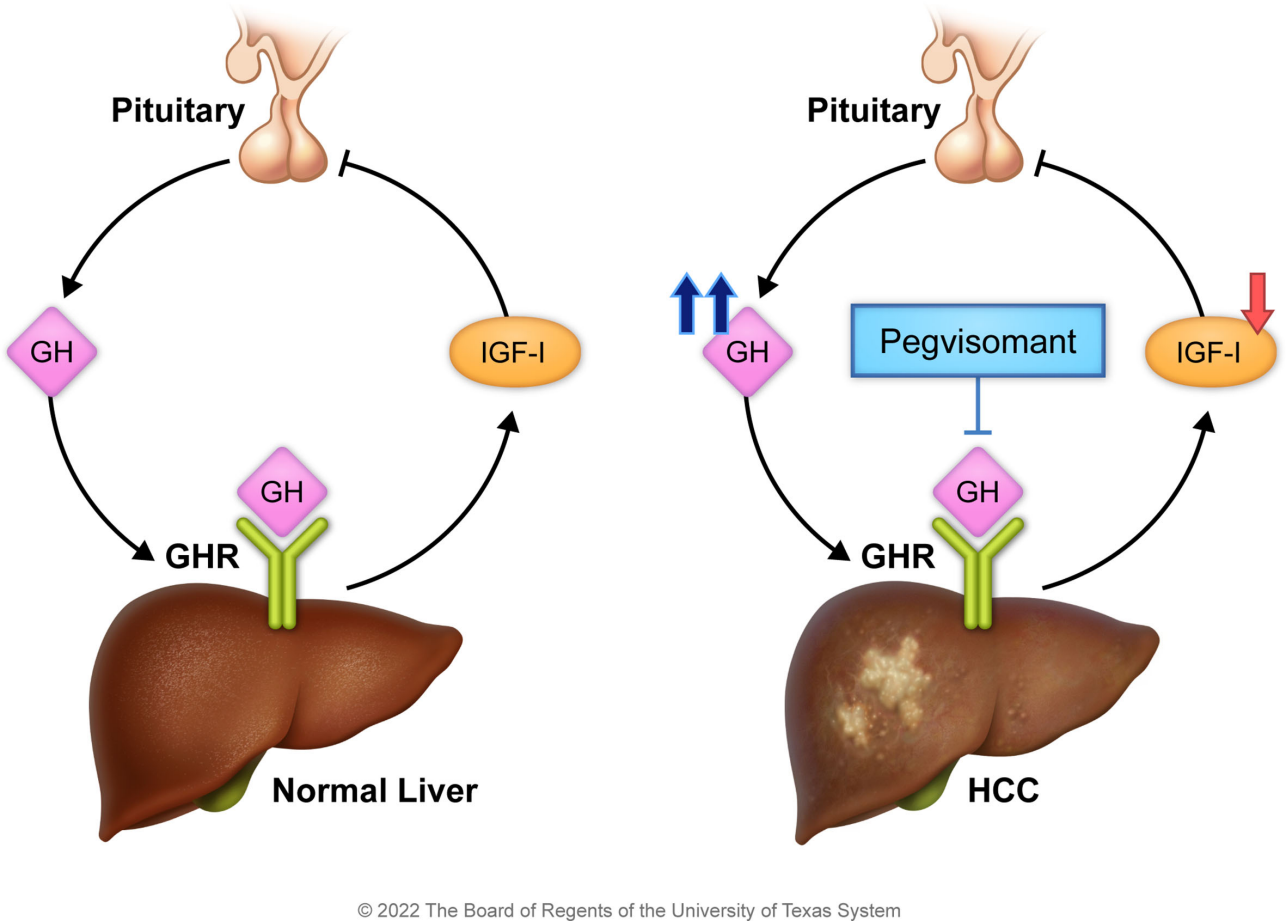
Working hypothesis and proposed model. Under the physiologic conditions (left panel), the pituitary secretes GH that activates hepatic GHR to stimulate IGF-I release. In turn, IGF-I suppresses the release of GH from the pituitary. In HCC (right panel), hepatic secretion of IGF-I is reduced because of liver insult, and its negative feedback effect on GH production is diminished. The marked production of GH by the pituitary promotes HCC survival. Hence, antagonism of GH/GHR binding by pegvisomant, a specific blocker of GH/GHR signaling, suppresses HCC.

Previous preclinical and clinical observations suggest that GHR/GH signaling plays an important role in HCC pathogenesis (18, 21–28). However, a major gap still exists in current understanding of the relevance of this pathway to HCC and none of these observations has been translated into a clinical trial strategy to block GHR signaling in HCC patients. Our working hypothesis and proposed model **(** **Figure 1** **)** are that chronic liver disease and liver tissue insult decrease hepatic production of circulating IGF-I in HCC patients (28–30). Subsequently such a decrease leads to increased pituitary secretion of GH *via* loss of negative feedback from IGF-I. In turn, increased GH enhances the activation of GHR. These molecular events support the survival and progression of HCC and, therefore, counteracting these events by targeting GHR, e.g., by pegvisomant, may lead to antitumor effects. Of note is that pegvisomant is a small peptide that blocks the binding and interactions between GHR and GH and is currently used to treat acromegaly patients who suffer increased GH secretion and hyperactivity of GHR. To achieve our goals, we measured circulating GH levels in a large cohort of HCC patients and normal controls, and correlated the results with response to therapy and patients’ survival. We also studied the effects of specific antagonism of GHR signaling *in vitro* by using siRNA and pegvisomant. Moreover, we studied the effects of pegvisomant *in vivo* using parental and sorafenib-resistant HCC cells. Finally, we treated two patients with advanced HCC and elevated circulating GH, who developed tumor growth and resistance to sorafenib, with pegvisomant. Our results show that GH/GHR signaling pathway plays important roles in HCC pathogenesis and suggest that exploiting this pathway may represent a successful therapeutic strategy for this aggressive neoplasm.

## Materials and methods

### Reagents

Human recombinant growth hormone (hrGH) was obtained from R&D Systems (catalogue number: 1067-GH-025; Minneapolis, MN). GHR siRNA was purchased from Santa Cruz Biotechnology (sc-40015; Santa Cruz, CA). Pegvisomant (B2036-PEG, Somavert; Pfizer, New York, NY) was dissolved in sterilized water and stored at -20°C until used. Sorafenib (Nexavar; Bayer, Whippany, NJ) stock solution (8×) was prepared by dissolving sorafenib powder in solution containing cremophor (MilliporeSigma, St. Louis, MO) and ethanol (1:1), with DMSO at <0.3% of the final volume. The stock solution was diluted with sterilized distilled water to 1× solution before being administered to the mice.

### Antibodies

The GHR antibodies were purchased from Santa Cruz Biotechnology (sc-137185) and Bioss Antibody (bs-0654R; Woburn, MA); the pJAK2^Y1007/Y1008^ (3771), JAK2 (3230), pSTAT3^Y705^ (4113), STAT3 (12640), pSTAT5^Y694^ (9314), STAT5 (9363), pIRS-1^S639^ (2388), IRS-1 (2382), pAKT^S473^ (4051), pERK1/2^T202/Y204^ (4370), c-Myc (5605), and Ki-67 (9027) antibodies were purchased from Cell Signaling Technology (Danvers, MA); BCL-xS/L (sc-1041), and MCL-1 (sc-819) were purchased from Santa Cruz Biotechnology; pIGF-IR^Y1161^ (ab39398) was purchased from AbCam (Cambridge, MA); and β-actin (A2228) was purchased from MilliporeSigma.

### Cell lines and normal human liver cells

The cell lines HepG2, SNU-387, SNU-423, and SNU-475 were purchased from ATCC (Manassas, VA). HepG2 cells were cultured in DMEM high-glucose medium (MilliporeSigma), and SNU-387, SNU-423, and SNU-475 cells in RPMI-1640 (MilliporeSigma). Culture media were supplemented with 10% fetal bovine serum, 100 U/mL penicillin, and 100 µg/mL streptomycin. Cells were grown at 37°C in 5% CO_2_. Normal human liver cells (ABM-T0051; Applied Biological Materials, Richmond, BC, Canada) were stored at -80°C. Before use, the liver cells were quickly thawed in a 37°C water bath. The resulting cell suspension was transferred into cold culture medium (Hepatocyte Culture Medium BulletKit [CC-3198]; Lonza, Walkersville, MD) and then centrifuged at 150*g* for 5 minutes at 4°C. Thereafter, the cell pellet was suspended in hepatocyte culture medium.

### Sorafenib-resistant HepG2 cells

Parental HepG2 cells were cultured in increasing concentrations of sorafenib in a humidified incubator at 37°C in 5% CO_2_. To determine the IC_50_, cell viability was measured by using the MTS assay. HepG2 cells were then cultured with sorafenib at a concentration just below the IC_50_. Sorafenib concentration was gradually increased by 0.25 µM/L per week. After 16-20 weeks, sorafenib resistance was assessed by comparing the cell viability of HepG2-SR cells with that of HepG2 cells. HepG2-SR cells were maintained in DMEM high-glucose medium supplemented with 5.0 µM sorafenib.

### Cell viability

Cells were seeded in 96-well cell culture plates and incubated overnight before serum starvation for 24 hours. Cells were then treated with hrGH (1.0 µg/mL) with or without pegvisomant. To measure the viability of the cells, 20 µL of the MTS reagent 3-(4, 5-dimethylthiazol-2-yl)-5-(3carboxymethoxyphenyl)-2-(4-sulfophenyl)-2H-tetrazolium (Promega, Madison, WI) was added to each well and cells were incubated for 1 hour at 37°C in 5% CO_2_. Absorbance at 490 nm was detected using a plate reader (CLARIOstar; BMG Labtech, Cary, NC).

### Cell proliferation

A standard kit (X1327K1; Exalpha Biologicals, Shirley, MD) was used to measure cell proliferation. Briefly, cells were incubated for 24 hours in 20 µL of BrdU (1:500). Then, 100 µL of anti-BrdU antibody was added for 1 hour, followed by 100 µL of peroxidase goat anti-mouse IgG conjugate (1:2000) for 30 minutes and 100 µL of TMB substrate for another 30 minutes. Fifty microliters of stop solution was added to stop the reaction, and absorbance was measured at 450/595 nm.

### Cell adhesion

Adhesion assays were performed in 12-well plates coated with extracellular matrix gel from Engelbreth-Holm-Swarm murine sarcoma cells (ECM Gel [E1270]; MilliporeSigma) diluted 2-fold using appropriate cell culture media. Control cells and cells treated with GHR inhibitor were added to the gel-coated plates and incubated for 72 hours. After incubation, the plates were washed with phosphate-buffered saline (PBS) to remove the unattached cells. Attached cells were then stained with 0.5% crystal violet for 1 hour at room temperature. After the wells were washed with PBS, the bound cells were evaluated by using bright-field microscopy (magnification: ×25).

### Cell migration

Cell migration was analyzed using 24-well Corning Transwell plates with polycarbonate membranes (3428; MilliporeSigma). Briefly, control cells or cells treated with GHR inhibitor in serum-free culture medium were loaded into the upper compartment and serum-free culture medium with 1.0 µg/mL hrGH was simultaneously loaded into the lower compartment. The plates were incubated for 4 hours at 37°C, and a particle counter and size analyzer (Beckman Coulter, Brea, CA) was used to count the cells that migrated through the membrane into the lower chamber.

### Anchorage-independent colony formation

Cells were plated in methylcellulose-based medium (Methcoult H4230; Stemcell Technologies, Vancouver, BC, Canada) mixed with DMEM or RPMI-1640 (1:5). Thereafter, the cells were harvested, suspended in methylcellulose (1:10), poured into 24-well plates, and incubated for 5 days at 37°C in 5% CO_2_. Cell colonies were stained using p-iodonitrotetrazolium violet and visualized using the FluorChem 8800 imaging system (Alpha Innotech, San Leandro, CA).

### Western blotting

Whole cell lysates were extracted using lysis buffer. Protein concentrations were determined using a protein assay kit (Quick Protein Assay Kit [500-0006]; Bio-Rad, Hercules, CA). Equal amounts of protein were separated using 12% sodium dodecyl sulfate–polyacrylamide gel electrophoresis and transferred to a polyvinylidene difluoride membrane (Millipore, Burlington, MA). Membranes were incubated with appropriately diluted specific primary antibodies. Protein bands were detected with an enhanced chemiluminescence kit (32106; ThermoFisher Scientific, Waltham, MA).

### Immunohistochemistry and immunofluorescence staining

Formalin-fixed and paraffin-embedded tissue sections from the tumor xenografts were subjected to xylene and alcohol gradient deparaffinization. Briefly, the sections were washed and then placed in a steamer with 1× target retrieval solution (Dako, Carpinteria, CA) for 25 minutes, allowed to cool for 20 minutes at room temperature, washed, and incubated in 3% H_2_O_2_ for 25 minutes to block endogenous peroxidase activity. The sections were then washed three times and incubated for 30 minutes in serum-free blocking solution (Dako) at room temperature. Primary antibodies (Ki-67, 1:400; GHR, 1:100; pAKT^S473^, 1:10; pERK1/2^T202/Y204^, 1:50; pIGF-IR^Y1161^, 1:75) were diluted in blocking buffer and added for overnight incubation at 4°C. Next, the sections were washed three times and incubated with the secondary antibody (K4063; Envision Dual Link HRP System; Dako) for 30 minutes. The slides were washed three times and developed with 3,3´-Diaminobenzidine substrate using the Dako chromogen system (K3468). Hematoxylin and eosin (H&E) were used for counterstaining. To calculate the proliferation index (PI), the Ki-67-positive cells in at least 10 high-power fields (×400) were counted, and the average number of positive cells per 1 high-power field was calculated. The light photomicrographs were captured using an Olympus BX41 microscope (Olympus Scientific Solutions, Waltham, MA), Infinity 3 camera (Teledyne Lumenera, Ottawa, Ontario, Canada), and Infinity Capture software (version 6.3.2., Teledyne Lumenera). The fluorescence photomicrographs were captured using an Olympus 1X70 fluorescence microscope (Olympus), Olympus DP72 camera (Olympus), and Aperio ImageScope software (Lecia Biosystems, Deer Park, IL).

### GH levels in HCC patients

Plasma samples from 767 HCC patients (567 males and 200 females) (489 with underlying cirrhosis and 278 without cirrhosis) admitted to our institution between 2001 and 2014 were analyzed for GH levels using the Rules-Based Medicine (RBM) multiplex ELISA (Myriad RBM, Austin, TX) in a CLIA-certified environment. Plasma from 200 healthy individuals (118 males and 82 females) was used as control.

### *In vivo* experiments

Animal experiments were approved by our Institutional Animal Care and Use Committee. Swiss nu-nu/Ncr nude mice (5-6 weeks old) were purchased from the Department of Experimental Radiation Oncology (authorized vendor, The University of Texas MD Anderson Cancer Center, Houston, TX). HepG2 or HepG2-SR cells (5.0 × 10^6^) dissolved in PBS and matrigel (1:1 in volume; 354234; Corning Life Sciences, Tewksbury, MA) were subcutaneously injected into the mice’s flanks. Approximately 7 days after inoculation, visible tumor growth was noted. Tumor length and width were measured using digital calipers, and tumor volumes were calculated using the formula: volume = π/6 × length × width^2^. When tumor volumes were between 70-90 mm^3^, mice were randomly divided into the different groups described in the results. Termination of the experiments was performed after 24 days or when signs of tumor burden developed. After euthanasia, tumors were dissected, weighed, and fixed in 10% buffered formalin for further immunohistochemical analysis. Tumor growth curves were plotted using average tumor volumes within each experimental group at the set time point.

### Enzyme-linked immunosorbent assay

Prior to euthanasia, blood was collected from the mice by cardiac puncture and then left to clot at room temperature for 30 minutes. Serum was separated by spinning the blood in a cooled Eppendorf centrifuge at 450*g* for 10 minutes. Serum GH and IGF-I levels were measured using mouse-specific ELISA kits from Biomatik (EKU04609; Wilmington, DE) and R&D Systems (MG100), respectively.

### Pegvisomant treatment of HCC patients

We treated two male patients with pegvisomant (10 mg/day). The patients had high plasma GH levels and developed clinical resistance to sorafenib leading to progressive disease. Pegvisomant was administered based on an off-label use of the FDA-approved drug owing to the patients’ high plasma GH levels.

### Statistical analysis

Statistical differences between the experimental groups were measured by using two-way ANOVA or Student’s *t*-test where appropriate in the *in vitro* studies and by using one-way ANOVA in the *in vivo* studies. Prism 8 for MacOS (GraphPad Software, San Diego, CA) was used for statistical analysis. Plasma biomarkers were summarized using descriptive statistics, and categorical clinical variables were tabulated with frequency and percentage. The Kruskal-Wallis test was applied for comparison of biomarkers among groups of healthy controls vs. HCC patients with or without cirrhosis, whereas Wilcoxon rank sum test was used for two-group comparisons including healthy controls vs. HCC, and HCC with cirrhosis vs. HCC without cirrhosis. Chi-square test was used to evaluate the association of GH level (high vs. normal) and categorical clinical factors. OS was estimated using the Kaplan-Meier method and compared using the log rank test. SAS 9.4 (SAS Institute, Cary, NC) and S-PLUS (v8.2; TIBCO Software, Palo Alto, CA) were used for statistical analysis. P<0.05 was considered statistically significant.

## Results

### High GH levels are associated with poorer clinical outcome in HCC patients

**Figure 2:**
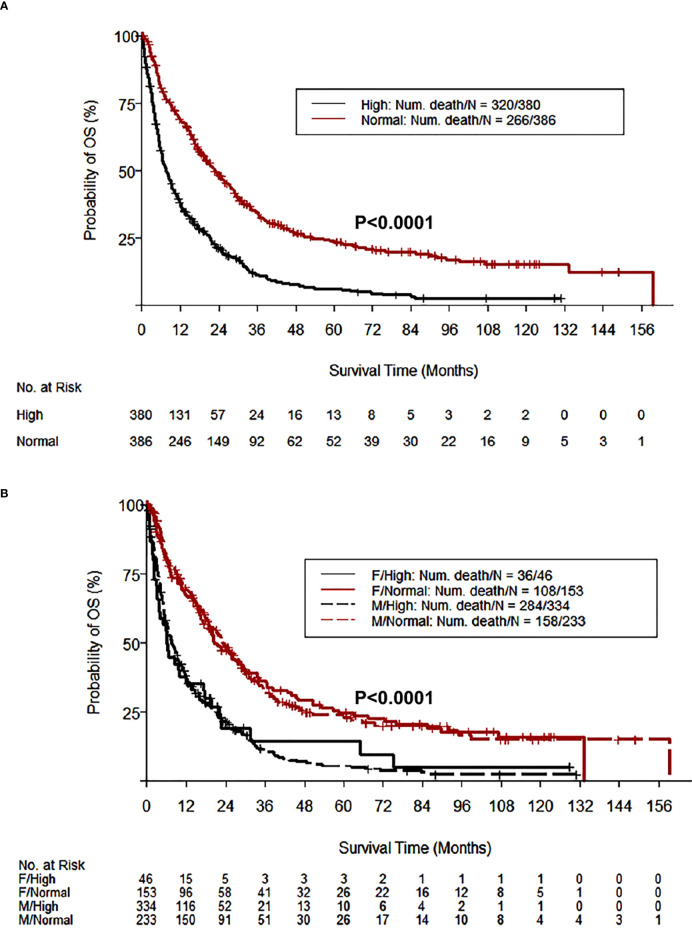
HCC patients with high GH levels have shorter OS. The Kaplan-Meier curves of OS according to GH levels demonstrate that 49.5% of HCC patients (380/767) with high GH levels (>0.97 and >3.7 µg/L in males and females, respectively) had a significantly shorter OS compared with patients with normal GH levels (P<0.0001) **(A)**. The shorter OS survival in HCC patients with high GH levels was gender-independent **(B)**.

Human studies were performed in accordance with the Declaration of Helsinki and approved by our Institutional Review Board. We measured plasma GH levels in 767 HCC patients (567 males, 200 females) and 200 healthy controls (118 males, 82 females). High GH levels (mean ± SD = 2.98 ± 4.4 µg/L) were found in 49.5% of HCC patients compared with low GH in 50.5% of the patients (0.41 ± 0.96 µg/L) (P<0.0001) (**Table 1**). High GH levels were associated with advanced disease and poor clinical outcome including more frequent cirrhosis, poorly differentiated tumors, multi-nodularity, vascular invasion, higher α-fetoprotein (AFP) levels, and advanced clinicopathological staging (**Table 1**). In agreement with our hypothesis and proposed model **(** **Figure 1** **)**, patients, irrespective of their gender, with high GH levels had significantly lower circulating IGF-I than patients with low GH levels (P<0.0001) **(** **Table 2** **)**. In addition, there was no difference in IGF-I levels in males vs. females when compared according to the GH levels **(** **Supplementary Table** **)**. Importantly, HCC patients with high GH had a shorter OS (median = 7.59 months, 95% confidence interval (CI): 6.34, 9.36) vs. patients with normal levels of GH (22.74 months, 95% CI: 19.52, 27.47) (P<0.0001) **(** **Figure 2A**; **Table 3** **)**. The shorter OS survival of HCC patients with high GH was gender-independent (**Figure 2B**; **Table 3**).

**Table 1:** Clinicopathological characteristics on HCC patients with low vs. high GH levels.

Covariates		Low GH^*^ 0.69 ± 0.78	High GH^*^ 5.31 ± 5.41	P values
		N = 387 (50.5%)	N = 380 (49.5%)	
Gender	Females	154 (39.8%)	46 (12.1%)	< 0.0001
	Males	233 (60.2%)	334 (87.9%)	
Underlying cirrhosis	No	170 (43.9%)	108 (28.4%)	< 0.0001
	Yes	217 (56.1%)	272 (71.6%)	
Severity of underlying cirrhosis	Child-Pugh A	248 (64.1%)	164 (43.2%)	< 0.0001
	Child-Pugh B	131 (33.9%)	168 (44.2%)	
	Child-Pugh C	8 (2.1%)	48 (12.6%)	
Pathological differentiation	Poorly differentiated	86 (22.2%)	102 (26.8%)	0.0084
	Well to moderately differentiated	225 (58.1%)	179 (47.1%)	
	Missing	76 (19.6%)	99 (26.1%)	
Tumor nodularity	Multinodular	216 (55.8%)	258 (67.9%)	0.0021
	Uni-nodular	156 (40.3%)	114 (30%)	
	Missing	15 (3.9%)	8 (2.1%)	
Vascular invasion	No	301 (77.8%)	223 (58.7%)	<0.0001
	Yes	85 (22%)	156 (41.1%)	
	Missing	1 (0.3%)	1 (0.3%)	
Tumor involvement	≤50%	304 (81.9%)	258 (69.5%)	0.0001
	>50%	67 (18.1%)	113 (30.5%)	
AFP (ng/mL)	<400	289 (74.7%)	227 (59.7%)	< 0.0001
	≥400	98 (25.3%)	153 (40.3%)	
CLIP staging	Stage 0-2	294 (76%)	191 (50.3%)	< 0.0001
	Stage 3	53 (13.7%)	94 (24.7%)	
	Stage 4-6	24 (6.2%)	85 (22.4%)	
	Missing	16 (4.1%)	10 (2.6%)	
Okuda staging	Stage I	243 (62.8%)	142 (37.4%)	< 0.0001
	Stage II	123 (31.8%)	205 (53.9%)	
	Stage III	5 (1.3%)	24 (6.3%)	
	Missing	16 (4.1%)	9 (2.4%)	
TNM staging	Stage I-II	171 (44.2%)	82 (21.6%)	< 0.0001
	Stage IIIA	86 (22.2%)	139 (36.6%)	
	Stage IIIC	119 (30.7%)	147 (38.7%)	
	Missing	11 (2.8%)	12 (3.2%)	
BCLC staging	Stage 0-B	113 (29.2%)	59 (15.5%)	< 0.0001
	Stage C-D	268 (69.3%)	320 (84.2%)	
	Missing	6 (1.6%)	1 (0.3%)	

^*^Normal GH level in males is ≤0.97 µg/L and in females is ≤3.7 µg/L. Men with GH > 0.97 µg/L and women with GH >3.7 µg/L were grouped as "High GH" group, otherwise grouped as "Low GH" group. GH levels are shown as means ± SD.

GH, growth hormone; N, number of patients; AFP, α-fetoprotein; CLIP, Cancer of Liver Italian Program; TNM, tumor size, number of lymph nodes, absence or presence of metastases; BCLC, Barcelona Clinic Liver Cancer. P values compare patients with normal GH levels vs. patients with high GH levels.

**Table 2:** Age and IGF-I levels (ng/ml) in HCC patients with low vs. high GH levels.

	Low GH^*^ 0.69 ± 0.78	High GH^*^ 5.31 ± 5.41	P value
All patients	N = 387 (50.5%)	N = 380 (49.5%)	
Age	62.9 ± 12.6 65.0 (21.0 - 91.0)	62.1 ± 10.9 61.0 (27.0 - 88.0)	0.0407
	
IGF-I	59.0 ± 35.4 47.9 (17.4 - 202.7)	45.57 ± 27.6 38.0 (13.2 - 219.9)	<0.0001
	
Males	N = 233 (41.1%)	N = 334 (58.9%)	
Age	63.0 ± 12.8 65.0 (21.0 - 91.0)	62.3 ± 10.8 61.0 (27.0 - 88.0)	0.0972
	
IGF-I	56.8 ± 35.3 45.2 (17.4 - 202.7)	45.8 ± 27.7 38.3 (13.2 - 219.9)	0.0001
	
Females	N = 154 (77.0%)	N = 46 (23.0%)	
Age	62.7 ± 12.4 65.0 (21.0 - 87.0)	60.5 ± 11.7 62.0 (34.0 - 82.0)	0.1917
	
IGF-I	62.3 ± 35.4 52.3 (18.8 - 193.9)	44.3 ± 27.1 34.8 (20.4 - 148.2)	0.002
	

^*^Normal GH level in males is ≤0.97 µg/L and in females is ≤3.7 µg/L. Men with GH >0.97 µg/L and women with GH >3.7 µg/L were grouped as "High GH" group, otherwise grouped as "Low GH" group. Data are shown as means ± SD. The median and range (in brackets) are shown below the mean of each parameter. The median and range of the GH levels are shown in **Supplementary Table 1**.

IGF-I, type I inulin-like growth factor; GH, growth hormone; N, number of patients.

**Table 3:** Log rank test to compare OS between HCC patients with high vs. low GH levels *.

Variables		N	Events	Median OS (95% CI)	OS Rate at 3 years (95% CI)	OS Rate at 5 Years (95% CI)	P value
	All patients	766	586	14.19 (11.96, 16.13)	0.23 (0.2, 0.27)	0.15 (0.13, 0.19)	
GH	H	380	320	7.59 (6.34, 9.36)	0.11 (0.08, 0.16)	0.06 (0.04, 0.1)	<0.0001
	L	386	266	22.74 (19.52, 27.47)	0.34 (0.29, 0.4)	0.24 (0.19, 0.29)	
GH/Gender	H/Females	46	36	6.14 (3.75, 17.58)	0.14 (0.06, 0.35)	0.14 (0.06, 0.35)	<0.0001
	L/Females	153	108	20.53 (18.86, 28.94)	0.36 (0.29, 0.46)	0.24 (0.18, 0.33)	
	H/Males	334	284	7.72 (6.47, 9.43)	0.11 (0.08, 0.16)	0.05 (0.03, 0.09)	
	L/Males	233	158	23.23 (17.48, 28.68)	0.33 (0.27, 0.4)	0.24 (0.18, 0.31)	

^*^Normal GH level in males is ≤0.97 µg/L and in females is ≤3.7 µg/L. Men with GH >0.97 µg/L and women with GH >3.7 µg/L were grouped as "High GH" group, otherwise grouped as "Low GH" group.

N, number of patients; GH, growth hormone; H, high; L, low; OS, overall survival; CI, confidence interval.

### GHR is highly expressed in HCC cell lines and primary tumors from patients

**Figure 3:**
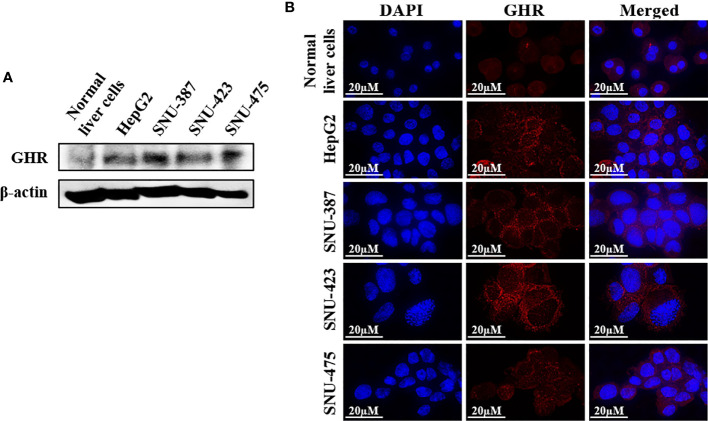
Expression of GHR in HCC cell lines. Western blotting **(A)** and immunofluorescence staining **(B)** demonstrate the expression of GHR in the cell lines HepG2, SNU-387, SNU-423, and SNU-475. The expression of GHR was markedly more pronounced in HCC cells than in normal human liver cells.

The association between high GH levels and poorer clinical outcome in HCC patients prompted additional investigation of the role of GH/GHR signaling in this aggressive neoplasm. Western blotting and immunofluorescence staining revealed that the expression of GHR in the HCC cell lines HepG2, SNU-387, SNU-423, and SNU-475 was upregulated compared to its level in normal human liver cells (**Figures 3A, B**). In addition, the expression of GHR and downstream target proteins including STAT3, STAT5, and IGF-IR was upregulated in primary human HCC tumors compared with the surrounding non-cancerous liver parenchyma and normal liver tissues (**Supplementary Figure 1**).

### Specific abrogation of GHR signaling by pegvisomant or siRNA induces pronounced inhibitory effects in HCC cell lines

**Figure 4:**
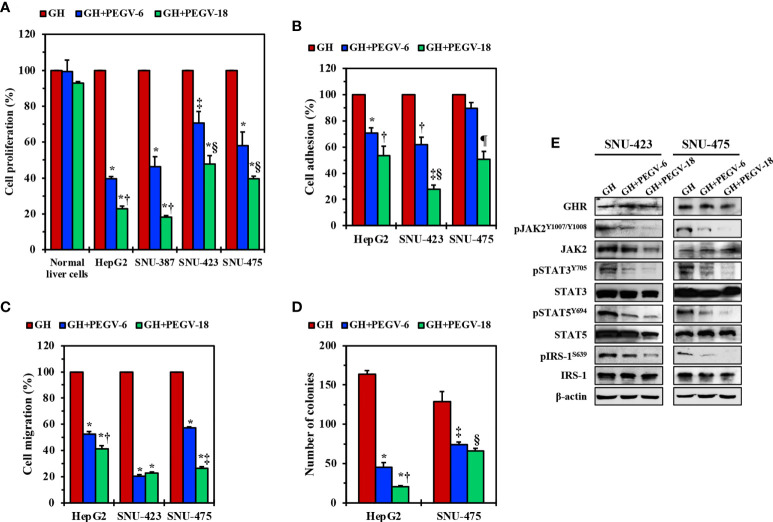
Effects of inhibition of GH/GHR signaling by using pegvisomant. In the presence of GH, pegvisomant (PEGV in the figure) at a concentration 6.0 or 18.0 µg/mL for 48 hours decreased cell proliferation in HepG2, SNU-387, SNU-423, and SNU-475 cells, which was not detected in normal liver cells **(A)** *P<0.0001 vs. GH; ^†^P<0.0001 vs. GH+PEGV-6; ^‡^P<0.01 vs. GH; ^§^P<0.05 vs. GH+PEGV-6. In addition, pegvisomant decreased the adhesion **(B)** *P<0.01, ^‡^P<0.001 vs. GH; ^‡^P<0.001 vs. GH+PEGV-6; ^§^P<0.0001 vs. GH; ^¶^P<0.01 vs. GH and GH+PEGV-6 and migration **(C)** *P<0.0001 vs. GH; ^‡^P<0.01 vs. GH+PEGV-6; ^‡^P<0.0001 vs. GH+PEGV-6 of HepG2, SNU-423, and SNU-475 cells. Furthermore, treatment of HepG2 and SNU-475 cells with pegvisomant reduced anchorage-independent colony formation potential **(D)** *P=0.0001 vs. GH; ^‡^P<0.05 vs. GH+PEGV-6; ^‡^P=0.01 vs. GH; ^§^P<0.01 vs. GH. Pegvisomant did not affect GHR levels of expression in SNU-423 and SNU-475 cells but significantly downregulated pJAK2, pSTAT3, pSTAT5, and pIRS-1 levels **(E)**.

Pegvisomant is a small peptide that specifically binds GHR and inhibits its binding with and activation by the GH. It was approved by the FDA to treat acromegaly (31, 32). To study the effects of pegvisomant in HCC cells, we cultured HepG2, SNU-387, SNU-423, and SNU-475 cells in FBS free medium, then stimulated these cells with GH in the presence or absence of pegvisomant at a concentration of 6 or 18 µg/mL for 48 h. Compared with control cells stimulated with GH alone, cell lines treated with pegvisomant (abbreviated PEGV in the figures) demonstrated decreased proliferation, adhesion, migration, and anchorage independent colony formation **(** **Figures 4A-D** **).** The effects of pegvisomant were dose-dependent and cells became more sensitive to its effect at the 18 µg/mL concentration. Consistent with its mechanism of action as a competitive blocker of GH binding with GHR, pegvisomant did not affect GHR expression in SNU-423 and SNU-475 cells (**Figure 4E**). Nonetheless, pegvisomant remarkably decreased the phosphorylation of JAK2, STAT3, STAT5, and IRS-1 (**Figure 4E**).

We also used another specific strategy, i.e., GHR siRNA, to further examine the effects of downregulation of GHR in HCC. Compared with scrambled siRNA, treating HepG2, SNU-423, and SNU-475 cells with GHR siRNA induced remarkable decreases in cellular viability, proliferation, adhesion, and migration (**Supplementary Figures 2A-D**). The effects of GHR siRNA were time-dependent and became more pronounced at 72 hours. Furthermore, transfecting HepG2 and SNU-475 cells with GHR siRNA significantly decreased their anchorage-independent colony formation potential after 5 days (**Supplementary Figure 2E** **)**. Mechanistically, siRNA-mediated downregulation of GHR decreased the phosphorylation of downstream effectors of GHR signaling including JAK2, STAT3, STAT5, and IRS-1 (**Supplementary Figure 2F**). Taken together, our pegvisomant and GHR siRNA *in vitro* data suggest that GH/GHR signaling pathway plays important roles in cell survival, growth, migration, and invasion.

### Combining pegvisomant and a low dose of sorafenib causes robust HCC tumor suppression *in vivo*

**Figure 5:**
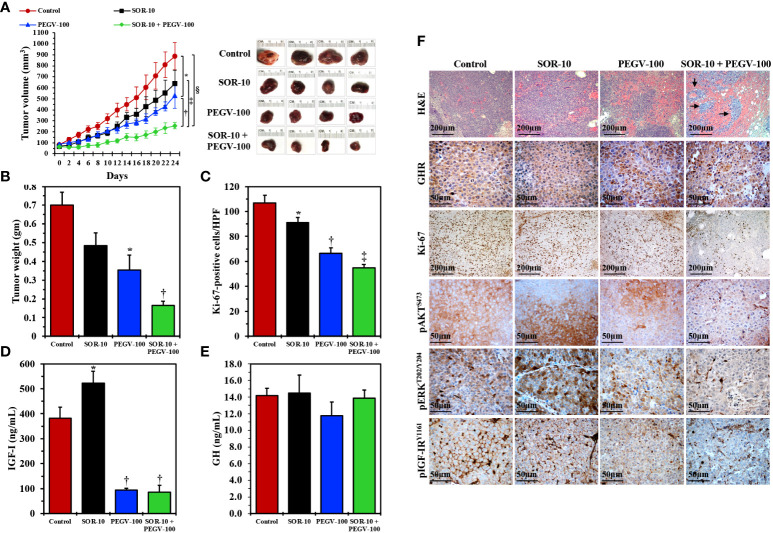
*In vivo* effects of antagonizing GH/GHR signaling axis using pegvisomant alone or in combination with a low dose of sorafenib in mice with parental HCC xenografts. Treating nude mice (females; control, seven mice; PEGV-100, seven mice; SOR-10, six mice; PEGV-100+SOR-10, six mice) harboring parental HepG2 HCC xenograft tumors with pegvisomant at a dose of 100 mg/kg/day (PEGV-100) significantly decreased the growth **(A)** Representative examples of HCC tumors from the four groups after necropsy on day 24 are shown in the right panel), weight **(B)**, and proliferation **(C)** of these tumors when compared to control mice treated with vehicle. In contrast, sorafenib alone at a dose of 10 mg/kg/day (SOR-10) did not have significant effects on tumor growth and weight and induced a very minimal decrease in cell proliferation. Of important note, the combined inhibitory effects of PEGV-100 and SOR-10 on HCC tumor growth and weight were superior to the effects of either of these two drugs alone (**A** *P = 0.02 vs. control mice; ^†^P = 0.02 vs. PEGV-100; ^†^P < 0.01 vs. SOR-10; ^§^P = 0.0002 vs. control, **B** *P < 0.01 vs. control; ^†^P < 0.05 vs. PEGV-100; P < 0.001 vs. SOR-10; P < 0.0001 vs. control, **C** *P = 0.04 vs. control; ^†^P < 0.0001 vs. control and P < 0.001 vs. SOR-10; ^‡^P < 0.0001 vs. control and SOR-10 and P = 0.02 vs. PEGV-100). Notably, the combined suppression became statistically significant only 8 days after treatment initiation (P < 0.05 vs. control). Whereas sorafenib alone significantly increased the level of circulating IGF-I, treating the mice with pegvisomant, alone or in combination with sorafenib, was associated with a remarkable decrease in IGF-I levels **(D)** *P < 0.001 vs. control; ^†^P < 0.001 vs. control and P < 0.0001 vs. SOR-10. Similar to its reported effects in humans, PEGV-100 treatment did not cause significant changes in GH levels in mice **(E)**. Histologically, H&E staining showed that control tumors or tumors treated with SOR-10 alone were composed of sheets of viable HCC cells, whereas the effects of PEGV-100 alone were more pronounced, in the form of larger hemorrhagic areas **(F)**. Importantly, the combined effects of PEGV-100 and SOR-10 were much more dramatic, in the form of expanded hemorrhagic areas that contained small islands of residual tumor cells (arrows) **(F)**. Immunohistochemical staining also revealed substantial levels of GHR expression in the four groups. However, in parallel with the changes depicted by H&E staining, immunohistochemical studies showed that the combination of PEGV-100 and SOR-10 significantly decreased tumor cell proliferation as detected by Ki-67 stain. However, the effects of dual treatment with PEGV-100 and SOR-10 on downregulating the expression of pAKT, pERK, and pIGF-IR were much more pronounced than the effects of the each of the two drugs as monotherapy **(F)**. Original magnification is ×100 for H&E and Ki-67 photomicrographs and ×400 for all other photomicrographs.

Next, we examined the effects of PEGV-100 and SOR-10, alone or in combination, on the growth of HepG2 xenograft tumors in nude mice. Tumor volumes were measured every 2 days and plotted against time. Whereas SOR-10 alone failed to induce significant suppression of tumor growth, PEGV-100 alone was capable of inhibiting tumor growth, and combining PEGV-100 with SOR-10 resulted in greater suppression of tumor growth (**Figure 5A**). We also measured tumor weights at the end of the study and found changes similar to those in tumor volumes (**Figure 5B**). The suppressive effects of the dual regimen on tumor growth were further illustrated in the form of a significant decrease in the PI as measured by Ki-67 staining. Whereas SOR-10 induced a minimal decrease in the PI, treatment with PEGV-100 was associated with a pronounced reduction (**Figure 5C**). Nonetheless, combined treatment with PEGV-100 and SOR-10 induced the largest decrease in the PI. We were also curious to know whether pegvisomant, similar to its effects in acromegaly patients, reduces circulating IGF-I levels in nude mice with HCC xenografts. Indeed, only mice treated with PEGV-100 alone or with PEGV-100 and SOR-10 had a significant decrease in circulating IGF-I levels (**Figure 5D**). Similar to patients who receive pegvisomant, significant changes in circulating GH levels were not observed in the mice after treatment with PEGV-100 (**Figure 5E**).

We also studied the effects of SOR-10 and PEGV-100, alone or in combination, by analyzing the xenograft tumor tissues collected at necropsy (**Figure 5F**). H&E-stained sections from control-treated tumors as well as tumors treated with SOR-10 alone were composed of sheets of tumor cells, whereas PEGV-100-treated tumors had expanded hemorrhagic areas consistent with therapy effects, which became much more pronounced in tumors treated with PEGV-100 and SOR-10. High levels of expression of GHR were documented in the four groups and, as suspected, treatment with PEGV-100 did not affect the basal tumor levels of GHR. However, cells stained with Ki-67 were much less pronounced in mice that received PEGV-100, and this effect was even greater with combined PEGV-100 and SOR-10, providing further evidence that combining PEGV-100 and SOR-10 suppresses tumor growth more than either of the two therapies alone. Similar effects were noted for pAKT, pERK, and pIGF-IR. Treatment with SOR-10 alone had almost no effect on the expression of these proteins. In contrast, there was a moderate decrease in expression after treatment with PEGV-100, which became much more pronounced when PEGV-100 and SOR-10 were combined.

### Pegvisomant overcomes sorafenib resistance *in vivo*

Resistance to sorafenib is commonly encountered in HCC patients and is considered a major hurdle for its utilization in the clinic. We set to study whether pegvisomant is capable of potentiating sorafenib effects and overcoming its resistance. We developed sorafenib-resistant HepG2 cells (HepG2-SR) in which sorafenib IC_50_ doubled from 6.25 µM in parental HepG2 cells to 12.5 µM in HepG2-SR cells (**Supplementary Figure 3A**). Biochemical alterations consistent with cell survival, proliferation, and apoptosis resistance were observed in the HepG2-SR cells, including upregulation of the antiapoptotic proteins BCL-xL and MCL-1, and the survival promoting proteins pAKT, pERK, and c-Myc, and downregulation of the proapoptotic protein BCL-xS (**Supplementary Figure 3B**). Because of the significant biochemical changes in Hep-SR cells and their resistance to sorafenib, we elected to increase the dose of pegvisomant to 200 mg/kg/day (PEGV-200) in this set of experiments.

**Figure 6:**
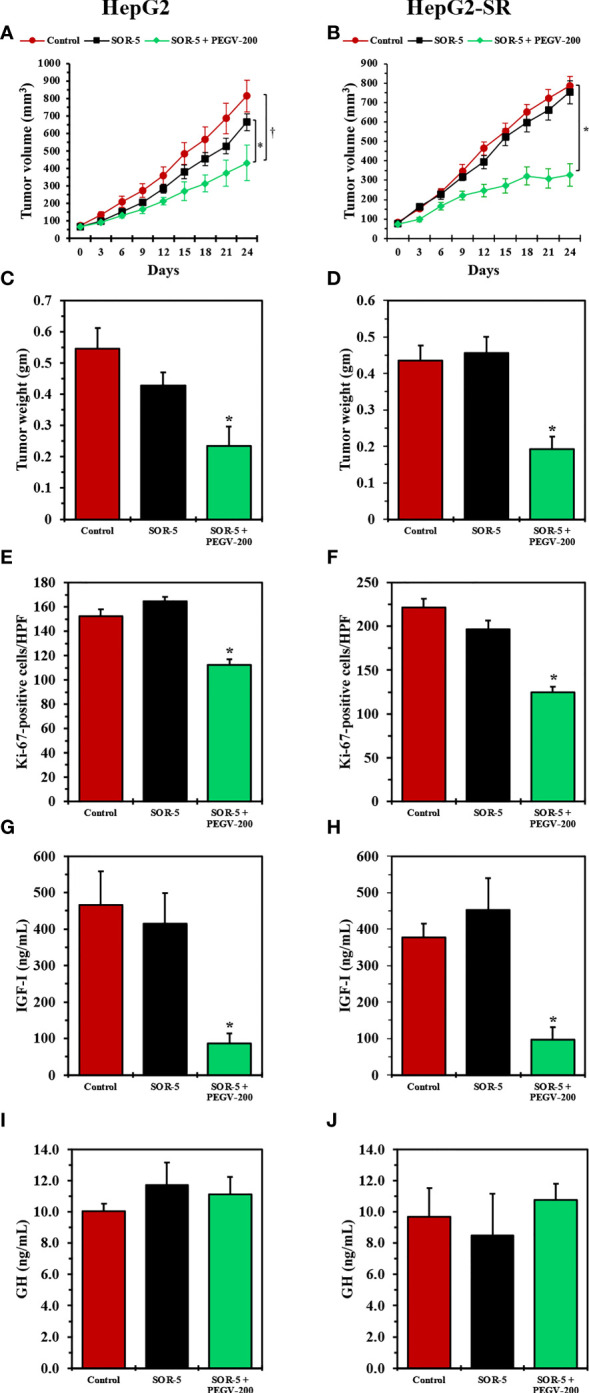
*In vivo* effects of antagonizing the GH/GHR signaling axis using pegvisomant in mice with sorafenib-resistant HCC xenografts. We studied the effects of SOR-5 alone vs. SOR-5+PEGV-200 in parental HepG2 tumor xenografts (left panel: five males, five females in the control group; four males, six females in the SOR-5 group; and five males, five females in the SOR-5+PEGV-200 group), and in the HepG2-SR tumor xenografts (right panel: five males, five females in the control group; three males, six females in the SOR-5 group; and six males, six females in the SOR-5+PEGV-200 group). In the parental HepG2 tumors, SOR-5 induced a slight decrease in tumor growth that was not statistically significant **(A)**, whereas in the sorafenib-resistant HepG2-SR tumors it failed to induce any suppression in tumor growth **(B)**. In contrast, treatment with PEGV-200 successfully suppressed the growth of these tumors **(A)** *P = 0.02 vs. SOR-5; ^†^P < 0.01 vs. control, **(B)** *P < 0.0001 vs. control and SOR-5. Similar effects were noted on the tumor weights **(C)** *P = 0.01 vs. SOR-5; P < 0.01 vs. control, **(D)** *P < 0.001 vs. control and SOR-5). Notably, SOR-5 alone didn’t decrease the proliferation of parental HepG2 tumor xenografts, whereas the addition of PEGV-200 induced a significant decrease in the proliferation of these tumors **(E, F)** *P < 0.0001 compared to control and SOR-5). In addition, only mice given PEGV-200 developed a marked decrease in circulating IGF-I levels **(G, H)** *P < 0.0001 vs. control and SOR-5). Treatment did not affect the levels of circulating GH **(I, J)**.

We used the HepG2-SR cells and the parental HepG2 cells, in parallel as a biological control, to develop tumor xenografts in nude mice. In parental HepG2 cells, sorafenib at a concentration of 5 mg/kg/day (SOR-5) slightly reduced tumors volumes (**Figure 6A**) and weights (**Figure 6C**). Of important note, the addition of pegvisomant PEGV-200 significantly enhanced the effects of SOR-5 (**Figures 6A, C**). Whereas in HepG2-SR cells SOR-5 had no inhibitory effect on tumor growth, treatment with PEGV-200 overcame sorafenib resistance and significantly decreased tumors volumes and weights (**Figures 6B, D**). In addition, treatment with PEGV-200 significantly decreased HCC cell proliferation, measured by Ki-67 proliferation index, in HepG2 and HepG2-SR tumor xenografts (**Figures 6E, F**). IGF-I is primarily secreted by the liver in response to activation of the GH/GHR signaling. In this study, treatment with PEGV-200, but not SOR-5, was associated with a dramatic decrease in circulating IGF-I levels both in HepG2 and HepG2-SR tumor bearing mice (**Figures 6G, H**). However, changes in GH levels did not associate the treatment with SOR-5 or SOR-5+PEGV-200 (**Figures 6I, J**). We also found that the ability of PEGV-200 to overcome sorafenib resistance was gender-independent because its effects on inhibiting tumor growth were similar in male and female mice (**Supplementary Figures 3C-F**).

The effects of pegvisomant were also studied in HepG2 **(** **Supplementary Figure 4**, left panel) and HepG2-SR **(** **Supplementary Figure 4**, right panel) tumor xenografts. H&E staining showed that control tumors as well as tumors treated with SOR-5 alone were composed of sheets of viable tumor cells. In contrast, treatment with PEGV-200 was associated with hemorrhagic areas and extensive tumor lysis consistent with therapeutic effects. High levels of GHR expression were seen in the HepG2 and HepG2-SR tumors; however, staining for Ki-67 illustrated that PEGV-200 significantly decreased the proliferation of the neoplastic cells. In addition, the administration of PEGV-200 dramatically downregulated the expressions of pAKT, pERK, and pIGF-IR in HepG2 and HepG2-SR tumor tissues, which became primarily localized in residual small clusters of tumor cells **(** **Supplementary Figure 4** **).**

### Pegvisomant induces tumor stability in HCC patients with clinical resistance to sorafenib

**Figure 7:**
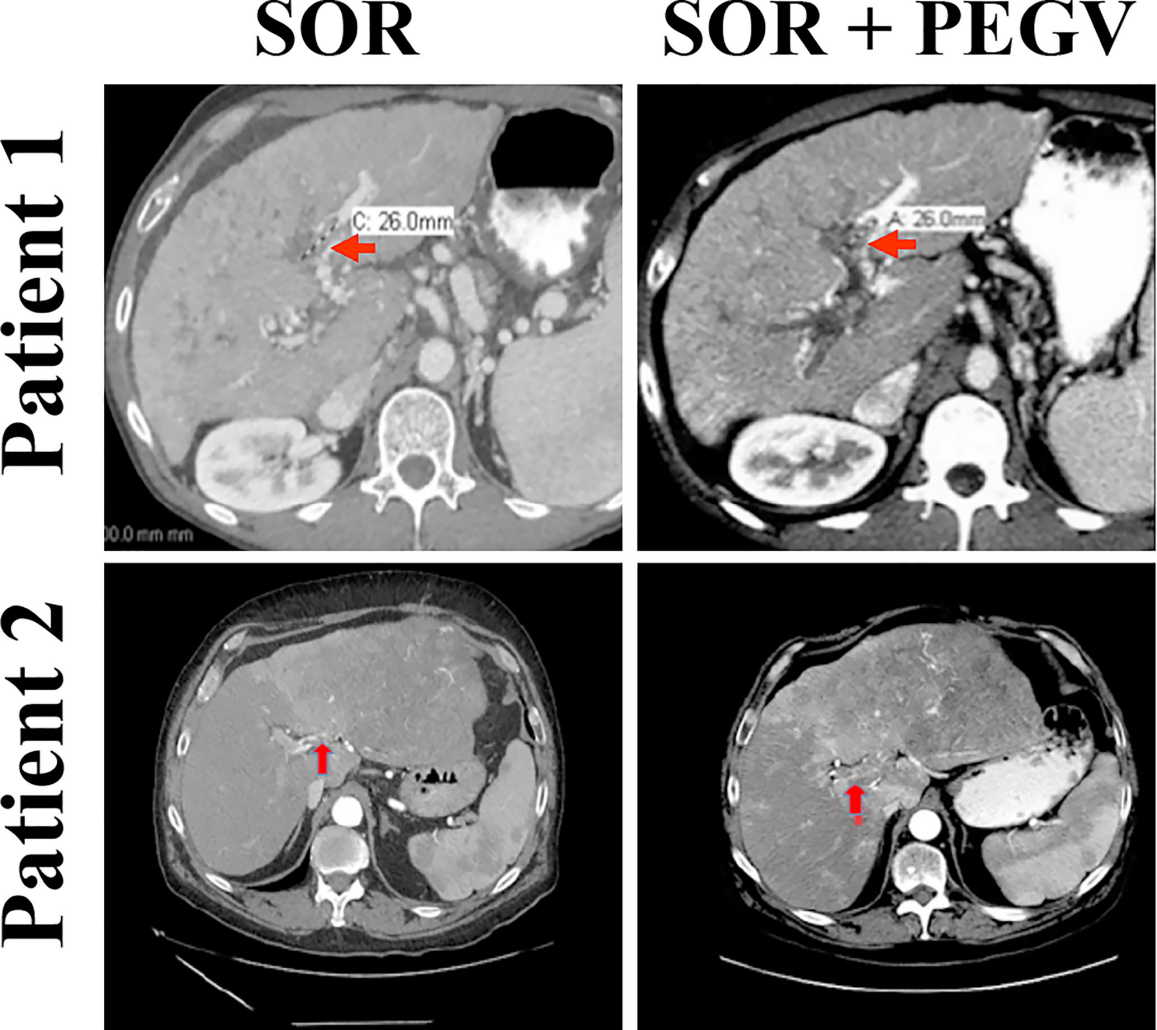
Blockade of GH/GHR signaling by pegvisomant causes tumor stability in HCC patients who develop sorafenib resistance. Two HCC male patients with clinical resistance and progression on sorafenib as well as with serum GH levels (5.3 and 3.7 µg/L) above those mandated by the FDA to allow administration of pegvisomant (normal level in males is ≤0.97 µg/L) were given pegvisomant, in addition to sorafenib, at a dose of 10 mg/kg/day subcutaneously. MRI scans after the addition of pegvisomant show disease stability. Patient 1 had a biomarker response in the form of a 40% decrease in AFP after combining pegvisomant with sorafenib. Patient 2 had low AFP (<20 µg/L) at baseline, which remained low on dual therapy.

Our *in vitro* and *in vivo* preclinical data suggest that targeting GH/GHR by using pegvisomant might represent a promising approach to treat HCC patients. Therefore, we treated two HCC patients who had high plasma GH levels with pegvisomant combined with sorafenib following clinical resistance and progression on sorafenib alone. Patient 1 was a 61-year-old white man with a history of alcoholic cirrhosis who had a large left liver HCC mass associated with left portal vein tumor thrombus (PVTT). This patient was treated with sorafenib. After 10 weeks, his AFP level had significantly increased, and imaging showed a new right PVTT. The patient’s GH levels were also elevated (5.3 µg/L), making him eligible for off-label administration of pegvisomant. Thus, pegvisomant (10 mg/day) was added to sorafenib at the time of progression. Patient 2 was a 54-year-old Hispanic man with hepatitis C-related cirrhosis and multifocal HCC who was also treated with sorafenib. He developed a new left PVTT about 6 months after sorafenib initiation and his AFP level was mildly elevated. Pegvisomant (10 mg/day) was added to sorafenib at the time of progression when he was found to have a high GH level (3.7 µg/L). Magnetic resonance imaging scans of the two patients showed PVTT thrombi prior to starting pegvisomant **(** **Figure 7**, left panel), and the same lesions stabilized upon administration of pegvisomant (after 6 weeks in patient 1 and 7 weeks in patient 2) **(** **Figure 7**, right panel). In addition to tumor stability, the administration of pegvisomant was associated with a 40% reduction in AFP levels in patient 1, while these levels remained stable in patient 2.

## Discussion

In this paper, we report that GH levels were significantly elevated in 49.5% of plasma samples from 767 HCC patients and that the elevated GH was associated with poorer clinical outcomes and advanced clinicopathological features including high AFP levels, underlying cirrhosis, multi-nodularity, and vascular invasion, as well as with advanced tumor size, number of lymph nodes, and presence of metastases (TNM) and Barcelona Clinic Liver Cancer (BCLC) staging systems. The association of high GH levels with poorer clinical outcomes was gender-independent, despite the notably higher physiological levels of circulating GH in women compared with men (normal GH level in women: ≤3.7 µg/L and in men: ≤0.97 µg/L). We also found that GHR is commonly overexpressed in HCC cell lines and in human tumors. Thereafter, we used pegvisomant to induce *in vitro* specific blockade of the GH/GHR binding and interaction, which resulted in decreased cell proliferation, adhesion, migration, and anchorage-independent colony formation of these cells, and mechanistically downregulated the phosphorylation of important downstream effectors of GHR, including JAK2, STAT3, STAT5, and IRS-1. The contribution of GHR signaling to the survival of HCC cells was further demonstrated when siRNA was used to specifically abrogate the expression of GHR. In the *in vitro* experiments we used cell lines with epithelial (HepG2) and mesenchymal (SNU-387, SNU-423, and SNU-475) phenotype. Pegvisomant and GHR siRNA induced significant inhibitory effects in these cell lines regardless of their phenotype background. Our *in vivo* experiments in nude mice showed that pegvisomant potentiated the effects of sorafenib and overcame sorafenib resistance in parental and sorafenib-resistant tumor xenografts, respectively. Pegvisomant decreased tumor growth in mice and significantly reduced tumor cell proliferation. Mechanistically, pegvisomant decreased the phosphorylation of ERK, AKT, and IGF-IR; downstream modulators of GH/GHR signaling that serve as survival proteins in HCC.

HCC is an aggressive malignancy with poor outcomes and limited curative treatment options for advanced-stage disease. The association between GH/GHR signaling and liver cell proliferation and the development of HCC has been previously demonstrated in different animal models. For instance, in GH-deficient dwarf rats, GH administration increases liver expression of genes that promote cell proliferation and survival, such as STAT3 and MAP kinase (33). Similarly, *Gh* transgenic mice suffer a remarkable increase in liver cell proliferation that leads to HCC, which has been attributed to higher GHR levels and increased expression and phosphorylation of survival-promoting targets including STAT3, ERK, AKT, EGFR, Src, and mTOR (21, 34, 35). Moreover, diethylnitrosamine-induced HCC was more frequently observed in *Gh* transgenic mice than in wild-type mice (22). Studies from different groups also demonstrated that GH/GHR signaling stimulates the proliferation of HCC cells *in vitro* and the growth of HCC tumor xenografts in nude mice (23–25). Recently, we demonstrated that downregulation of GHR expression hinders HCC development and decreases its tumor burden in mice with disrupted *Ghr* gene expression (26). In addition to animal-based evidence, previous studies also showed that GHR is highly expressed in human HCC tumors (27). In the current study, we demonstrated for the first time that specific blockade of GH/GHR signaling pathway using pegvisomant, an FDA-approved drug, potentiates the effects of sorafenib and overcomes its resistance in nude mice xenograft tumors as well as in two HCC patients who developed resistance and progressed on sorafenib. Collectively, these data suggest that GH/GHR signaling contributes to HCC pathogenesis and, therefore, that targeting GHR could be considered a potential approach to eradicate this aggressive neoplasm.

Pegvisomant is a genetically modified human GH analogue with several mutated amino acids (36). As a result, it binds to GHR dimer but does not induce the conformational changes required for signaling (37). Pegvisomant has been approved by the FDA to treat acromegaly, a clinicopathological state of elevated GH levels (31, 32). It is a highly specific drug because it does not cross-react with other structurally related receptors such as the prolactin receptor (38). Analyzing the pharmacodynamic characteristics of pegvisomant showed that up to 80 mg/day was tolerable with minimal adverse effects (39). Thus, the use of pegvisomant in HCC is advantageous because it is tolerable even at doses significantly higher than the 10 mg/day that we used in our patients, which could further enhance its anti-tumor activity. A few preclinical studies have investigated pegvisomant in malignant neoplasms and showed that it has promising effects in cancers of the colon, breast, and endometrium and in meningiomas (40–43). However, pegvisomant has not been systematically tested in patients with cancer. Our data present evidence that such an approach is feasible and applicable to routine practice. Of important note is that pegvisomant could also be combined with tyrosine kinase inhibitors or immunotherapy drugs that have been approved for HCC treatment, yet have limited effects.

When liver functions and reserve are normal, pegvisomant-mediated blockade of GH/GHR signaling results in decreased serum concentrations of total and free IGF-I, a growth-promoting cytokine in acromegaly and a survival-promoting cytokine for cancer cells, in humans (39). Similarly, we observed a remarkable decrease in circulating IGF-I levels in mice treated with pegvisomant. Unlike our nude mice with subcutaneously implanted HCC xenografts who maintain intact livers capable of secreting physiologic levels of IGF-I, patients with HCC encounter deterioration in liver reserve and function during disease progression, which leads to significant reduction in circulating IGF-I (28–30). In line with these observations, our current results show that IGF-I levels are significantly decreased in HCC patients who have high GH levels, owing to the loss of the negative feedback effect of IGF-I on GH secretion by the pituitary. Notably, patients with high GH/low IGF-I suffer a more advanced and aggressive disease and exhibit shorter OS. It is possible that IGF-I contributes to the survival of HCC during early stages. However, this effect wanes with disease progression, whereas GH/GHR signaling enhances to further promote the survival and progression of HCC. Additional studies are required to further explore this model.

In conclusion, our study tested and validated for the first time the concept of inhibiting GHR/GH signaling in HCC by using pegvisomant, a drug that is already FDA approved, at multiple levels including *in vitro* and *in vivo* preclinical experiments as well as a pilot human study that demonstrated activity in sorafenib-resistant settings. Altogether, the results support our hypothesis and proposed model shown in **Figure 1**. While we are aware that our therapeutic studies in patients represent a limited experience, these studies are relevant because they show potential clinical efficacy that warrants further investigation in future large, randomized, clinical trials by integrating pegvisomant into front- and second-line settings in HCC patients with high GH levels. We also anticipate that such an approach is advantageous and feasible given that approximately half of HCC patients have elevated GH levels and, therefore, could be eligible for pegvisomant treatment. Our findings may also lead to future studies exploring the targeting of GHR signaling in other aggressive neoplasms.

## Data availability statement

The original contributions presented in the study are included in the article/**Supplementary Material**. Further inquiries can be directed to the corresponding authors.

## Ethics statement

The studies involving human patients/participants were reviewed and approved by Institutional Review Board, The University of Texas MD Anderson Cancer Center. The patients/participants provided their written informed consent to participate in this study. The animal studies were reviewed and approved by Institutional Animal Care and Use Committee (IACUC), The University of Texas MD Anderson Cancer Center.

## Author contributions

AK and HA: conceptualization, resources, data curation, formal analysis, supervision, funding acquisition, validation, investigation, visualization, methodology, writing-original draft, project administration, and writing-review and editing. AH: data curation, formal analysis, validation, investigation, methodology, and writing-original draft. DV and BG: data curation, formal analysis, validation, investigation, and methodology. MH: resources, data curation, and formal analysis. LX and JM: formal analysis, validation, and methodology. VS, YM, RCP, JL, and RA: formal analysis and validation. JY, RW, and AR: resources and formal analysis. All authors contributed to the article and approved the submitted version.

## Funding

All of the authors declare no competing financial interests related to this work. This work was supported in part by the National Institutes of Health/National Cancer Institute grants R01CA151533 (HA), R21CA170035 (AK), R21CA190945 (AK), R01CA260872 (AK, HA), and by an MD Anderson Cancer Center Bridge Funding Grant (HA).

## Acknowledgments

We thank Ms. Dawn Chalaire for outstanding assistance with editing our manuscript, and Mr. Jordan Pietz for excellent help with generating **Figure 1**. Humanized pegvisomant was generously provided at no cost by Pfizer (New York, NY) for the mice experiments.

## Conflict of interest

The authors declare that the research was conducted in the absence of any commercial or financial relationships that could be construed as a potential conflict of interest.

## Publisher’s note

All claims expressed in this article are solely those of the authors and do not necessarily represent those of their affiliated organizations, or those of the publisher, the editors and the reviewers. Any product that may be evaluated in this article, or claim that may be made by its manufacturer, is not guaranteed or endorsed by the publisher.

## Supplementary material

Supplementary Table 1 Age and IGF-I levels (ng/ml) in male vs. female HCC patients.

Supplementary Figure 1 Expression of GHR in HCC primary human tumor tissues. Immunohistochemical staining was performed using primary HCC tumor samples from patients (9 males and three females). Both HCC tumors and the surrounding non-cancerous tissues (NCT) were stained. GHR was highly expressed in 10 (83%) out of 12 tumors. The expression of survival proteins that are known to interact with GHR, including STAT3, STAT5, and IGF-IR, was more pronounced in the HCC areas than in the surrounding NCT. Representative examples from two different tumors are shown in **(A)**. Weak expression of GHR, STAT3, STAT5, and IGF-IR was present in normal liver tissues. Three normal liver tissue samples are shown in **(B)**. Original magnification is ×400.

Supplementary Figure 2 Effects of specific downregulation of GHR by siRNA. HCC cell lines were transfected with scrambled siRNA or with GHR siRNA for 48 and 72 h. Compared with scrambled siRNA, GHR siRNA induced significant decrease in cell viability **(A)** *P < 0.0001 vs. scrambled siRNA; ^†^P < 0.05 vs. GHR siRNA [72 h]; ^‡^P < 0.001 vs. scrambled siRNA, proliferation **(B)** *P < 0.0001 vs. other conditions; ^†^P < 0.001 vs. scrambled siRNA; ^§^P < 0.0001 vs. scrambled siRNA; ^¶^P < 0.001 vs. GHR siRNA [48 h], adhesion **(C)** *P < 0.0001 vs. scrambled siRNA; ^†^P < 0.01 vs. GHR siRNA [72 h]; ^‡^P = 0.0001 vs. GHR siRNA [72 h]**)**, and migration **(D)** *P < 0.0001 vs. other conditions. GHR siRNA also decreased the anchorage-independent colony formation in HepG2 and SNU-475 cells **(E)** *P < 0.05 and ^†^P < 0.01 vs. scrambled siRNA. The results are shown as means ± SE. Transfection of GHR siRNA in HepG2, SNU-423, and SNU-475 cells decreased levels of GHR-interacting proteins pJAK2, pSTAT3, pSTAT5, and pIRS-1 **(F)**.

Supplementary Figure 3 The effects of pegvisomant on sorafenib-resistant HepG2 (HepG2-SR) xenografts are gender-independent. We used parental HepG2 cells to develop sorafenib-resistant counterparts (HepG2-SR). Cell viability assay shows that the IC_50_ for sorafenib doubled from 6.25 µM in parental HepG2 cells to 12.50 µM in HepG2-SR cells **(A)**. Despite that the development of sorafenib resistance in HepG2-SR cells was not associated with changes in GHR levels, sorafenib resistance was associated with pronounced alterations that favor cell survival including upregulation of BCL-xL and MCL-1 and downregulation of BCL-xS. Moreover, sorafenib resistance was associated with increased pAKT, pERK, and c-Myc **(B)**. We also found that the ability of PEGV-200 to overcome sorafenib resistance is gender independent because tumor growth and weight were similar in males (3 mice) and females (6 mice). In males: *P < 0.001 vs. control and SOR-5 for tumor growth **(C)** and: *P < 0.01 vs. control and SOR-5 for tumor weight **(D)**. In females: *P < 0.01 vs. control and SOR-5 for tumor growth **(E)** and tumor weight **(F)**.

Supplementary Figure 4 Pegvisomant overcomes sorafenib resistance and decreases tumor cell proliferation and the phosphorylation of downstream survival effectors of GH/GHR signaling in HepG2-SR tumor xenografts. H&E staining demonstrates that PEGV-200 treatment was associated with expanded areas of hemorrhage and tumor lysis (black circles) in HepG2 (left panel) and HepG2-SR (right panel) tumor xenografts. Admixed within these areas were small nests of residual tumor cells (black arrows). Immunohistochemical staining shows expression of GHR in tumor xenografts, however, Ki-67+ cells decreased remarkably in tumors from mice treated with PEGV-200 vs. those from control mice and mice treated with SOR-5 alone. The arrows highlight the small foci of residual tumor cells. In addition, pAKT, pERK, and pIGF-IR proteins were almost entirely lacking within the expanded areas of tumor hemorrhage and lysis and were limited only to scattered clusters of residual viable tumor cells in mice treated with PEGV-200 (arrows). Conversely, control tumors and tumors treated with SOR-5 alone showed high levels of expression of the activated forms of these survival proteins. Original magnification is ×100 for H&E, Ki-67, and the SOR-5+PEGV-200 photomicrographs and ×400 for all other photomicrographs.

## Glossary

**Table:**

	
AFP	Alpha-fetoprotein
AKT	Ak strain transforming
ANOVA	analysis of variance
ATCC	American Type Culture Collection
BCL-xS/L	B-cell lymphoma-extra small/large
BCLC	Barcelona Clinic Liver Cancer
BrdU	bromodeoxyuridine
CI	confidence interval
CLIP	Cancer of the Liver Italian Program
c-Myc	cellular myelocytomatosis virus
DMEM	Dulbecco’s modified Eagle medium
DMSO	dimethyl sulfoxide
EGFR	epidermal growth factor receptor
ELISA	enzyme-linked immunosorbent assay
ECM	extracellular matrix
ERK	extracellular signal-regulated kinase
FDA	Food and Drug Administration
GH	growth hormone
GHR	growth hormone receptor
H	high
HCC	hepatocellular carcinoma
HepG2-SR	sorafenib-resistant HepG2 cells
HR	hazard ratio
hrGH	human recombinant growth hormone
IGF-I	type I insulin-like growth factor
IGF-IR	type I insulin-like growth factor receptor
IRS-1	insulin receptor substrate 1
JAK2	Janus kinase 2
OS	overall survival
MAP kinase	mitogen-activated protein kinase
MCL-1	myeloid cell leukemia 1
mTOR	mammalian target of rapamycin
N	normal
No.	number of patients
PBS	phosphate buffered saline
PD-L1	programmed cell death-ligand 1
PEGV	pegvisomant
PFS	progression-free survival
PVTT	portal vein tumor thrombus
RPMI	Roswell Park Memorial Institute
siRNA	small interfering RNA
SOR	sorafenib
STAT	signal transducer and activator of transcription
TKI	tyrosine kinase inhibitor
TNM	tumor size, number of lymph nodes, and presence of metastases
VEGF	vascular endothelial growth factor
	
