# Supplementary material for: Blockade of growth hormone receptor signaling by using pegvisomant: A functional therapeutic strategy in hepatocellular carcinoma
Source: Front Oncol. 2022 Oct 6;12:986305. doi: 10.3389/fonc.2022.986305 (PMC9582251; doi:10.3389/fonc.2022.986305)
Supplement: Supplementary Table 1 — Age and IGF-I levels (ng/ml) in male vs. female HCC patients. [file Table_1.docx]

| **Supplementary Table: Age and IGF-I levels (ng/ml) in male vs. female HCC patients** | | | |
| --- | --- | --- | --- |
|  | **Females (N = 200)** | **Males (N = 567)** | **P value** |
| **Low GH^*^**  **0.69 ± 0.78**  **0.42 (0.1 - 3.6)** | **N = 154 (77.0%)** | **N = 233 (41.1%)** |  |
| Age | 62.7 ± 12.4  65.0 (21.0 - 87.0) | 63.0 ± 12.8  65.0 (21.0 - 91.0) | **0.9597** |
|  |  |  |  |
| IGF-I | 62.3 ± 35.4  52.3 (18.8 - 193.9) | 56.8 ± 35.3 | **0.1143** |
|  |  | 45.2 (17.41 - 202.7) |  |
| **High GH^*^**  **5.31 ± 5.41**  **3.5 (1.0 - 36.0)** | **N = 46 (23.0%)** | **N = 334 (58.9%)** |  |
| Age | 60.5 ± 11.7  62.0 (34.0 - 82.0) | 62.3 ± 10.8  61.0 (27.0 - 88.0) | **0.5289** |
|  |  |  |  |
| IGF-I | 44.3 ± 27.1  34.8 (20.4 - 148.2) | 45.8 ± 27.7  38.3 (13.2 - 219.9) | **0.6743** |
|  |  |  |  |

^*^Normal GH level in males is ≤0.97 µg/L and in females is ≤3.7 µg/L. Men with GH >0.97 µg/L and women with GH >3.7 µg/L were grouped as "High GH" group, otherwise grouped as "Low GH" group. Data are shown as means ± SD. The median and range (in parentheses) are shown below the mean of each parameter.

IGF-I, type I inulin-like growth factor; N, number of patients; GH, growth hormone.
