# Supplementary figures and images for: Blockade of growth hormone receptor signaling by using pegvisomant: A functional therapeutic strategy in hepatocellular carcinoma
Source: Front Oncol. 2022 Oct 6;12:986305. doi: 10.3389/fonc.2022.986305 (PMC9582251; doi:10.3389/fonc.2022.986305)

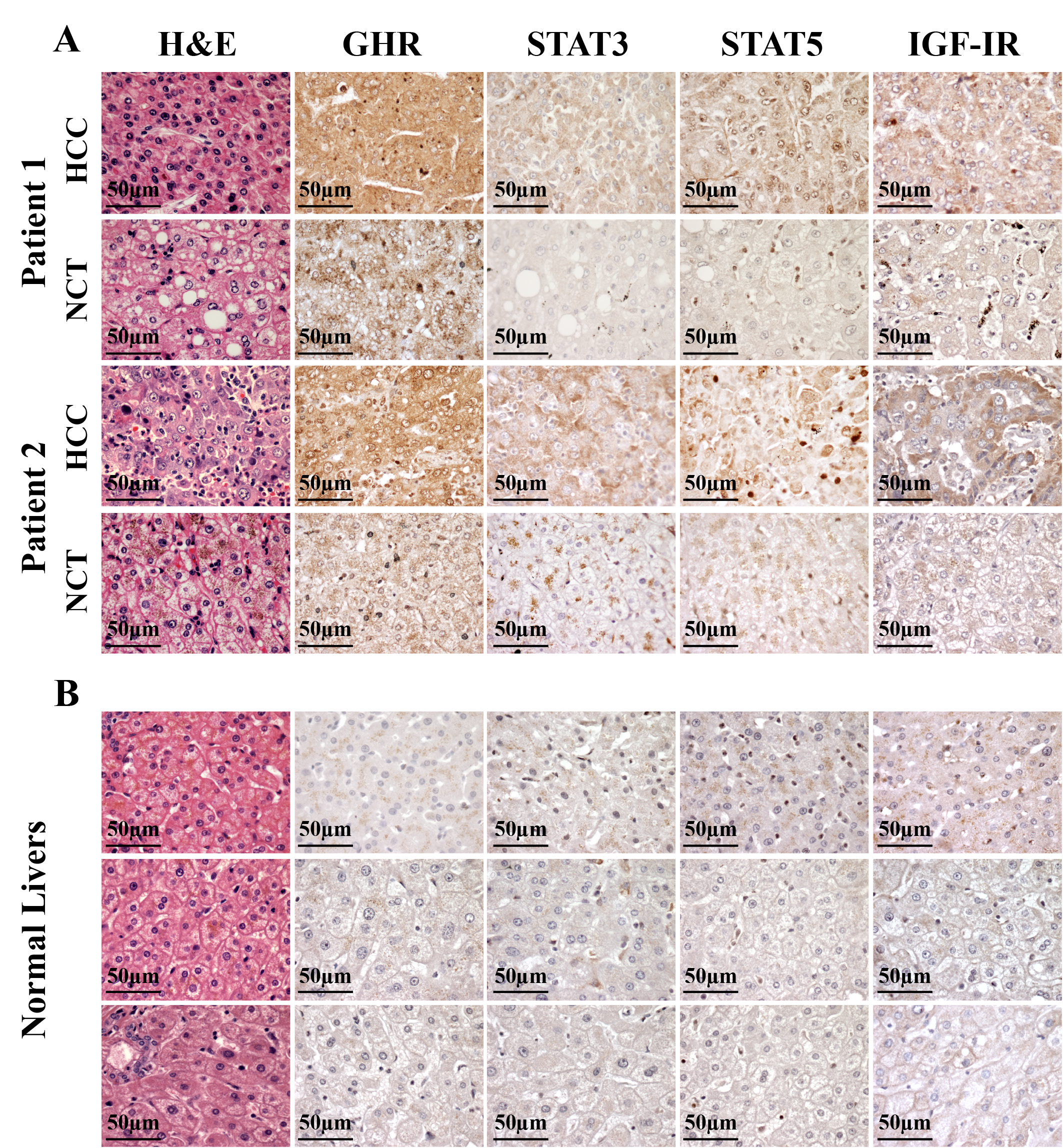

Supplement: Supplementary Figure 1 — Expression of GHR in HCC primary human tumor tissues. Immunohistochemical staining was performed using primary HCC tumor samples from patients (9 males and three females). Both HCC tumors and the surrounding non-cancerous tissues (NCT) were stained. GHR was highly expressed in 10 (83%) out of 12 tumors. The expression of survival proteins that are known to interact with GHR, including STAT3, STAT5, and IGF-IR, was more pronounced in the HCC areas than in the surrounding NCT. Representative examples from two different tumors are shown in (A). Weak expression of GHR, STAT3, STAT5, and IGF-IR was present in normal liver tissues. Three normal liver tissue samples are shown in (B). Original magnification is ×400. [file Image_1.tif]

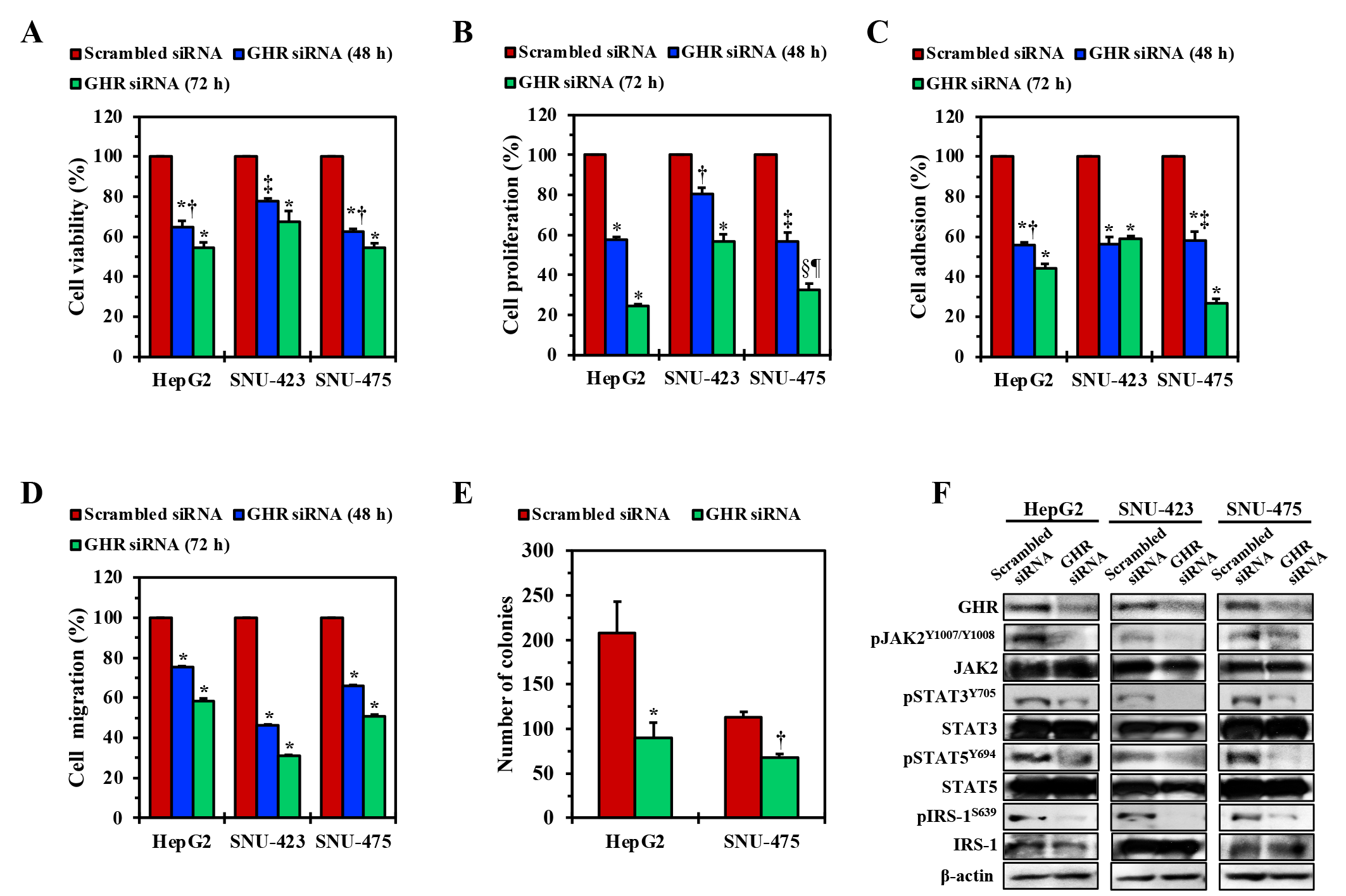

Supplement: Supplementary Figure 2 — Effects of specific downregulation of GHR by siRNA. HCC cell lines were transfected with scrambled siRNA or with GHR siRNA for 48 and 72 h. Compared with scrambled siRNA, GHR siRNA induced significant decrease in cell viability (A) *P < 0.0001 vs. scrambled siRNA; †P < 0.05 vs. GHR siRNA [72 h]; ‡P < 0.001 vs. scrambled siRNA, proliferation (B) *P < 0.0001 vs. other conditions; †P < 0.001 vs. scrambled siRNA; §P < 0.0001 vs. scrambled siRNA; ¶P < 0.001 vs. GHR siRNA [48 h], adhesion (C) *P < 0.0001 vs. scrambled siRNA; †P < 0.01 vs. GHR siRNA [72 h]; ‡P = 0.0001 vs. GHR siRNA [72 h]), and migration (D) *P < 0.0001 vs. other conditions. GHR siRNA also decreased the anchorage-independent colony formation in HepG2 and SNU-475 cells (E) *P < 0.05 and †P < 0.01 vs. scrambled siRNA. The results are shown as means ± SE. Transfection of GHR siRNA in HepG2, SNU-423, and SNU-475 cells decreased levels of GHR-interacting proteins pJAK2, pSTAT3, pSTAT5, and pIRS-1 (F). [file Image_2.tif]

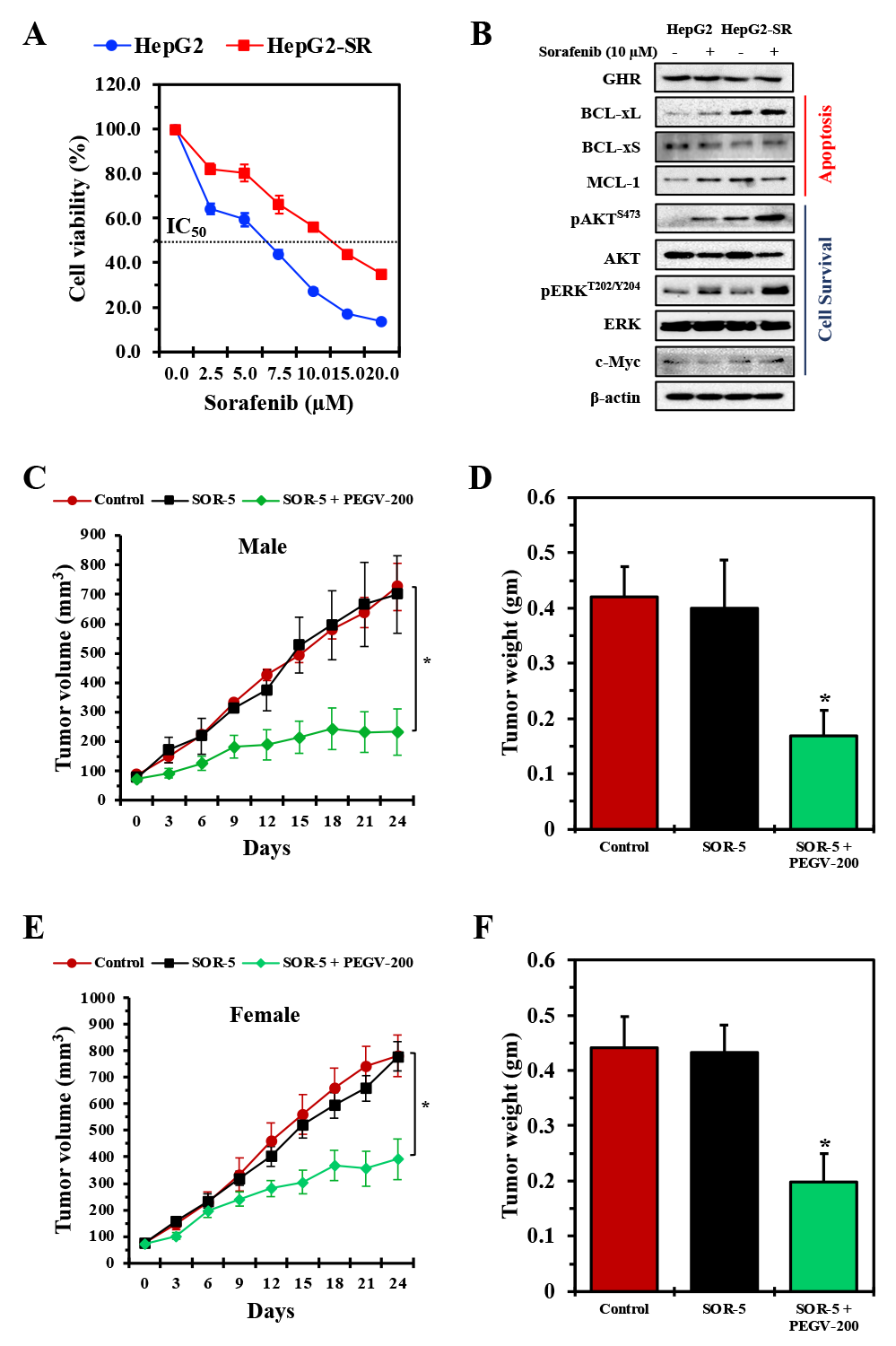

Supplement: Supplementary Figure 3 — The effects of pegvisomant on sorafenib-resistant HepG2 (HepG2-SR) xenografts are gender-independent. We used parental HepG2 cells to develop sorafenib-resistant counterparts (HepG2-SR). Cell viability assay shows that the IC50 for sorafenib doubled from 6.25 µM in parental HepG2 cells to 12.50 µM in HepG2-SR cells (A). Despite that the development of sorafenib resistance in HepG2-SR cells was not associated with changes in GHR levels, sorafenib resistance was associated with pronounced alterations that favor cell survival including upregulation of BCL-xL and MCL-1 and downregulation of BCL-xS. Moreover, sorafenib resistance was associated with increased pAKT, pERK, and c-Myc (B). We also found that the ability of PEGV-200 to overcome sorafenib resistance is gender independent because tumor growth and weight were similar in males (3 mice) and females (6 mice). In males: *P < 0.001 vs. control and SOR-5 for tumor growth (C) and: *P < 0.01 vs. control and SOR-5 for tumor weight (D). In females: *P < 0.01 vs. control and SOR-5 for tumor growth (E) and tumor weight (F). [file Image_3.tif]

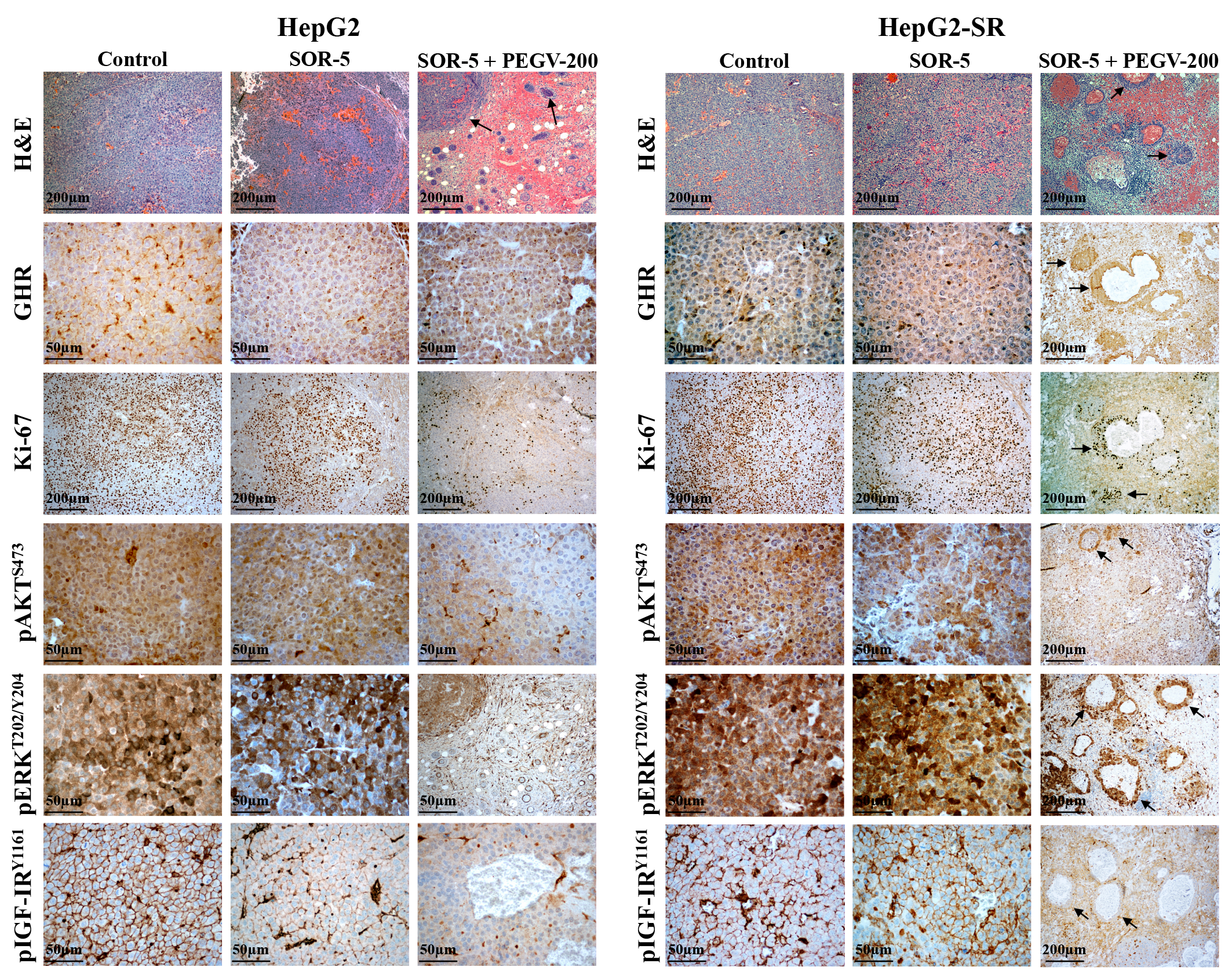

Supplement: Supplementary Figure 4 — Pegvisomant overcomes sorafenib resistance and decreases tumor cell proliferation and the phosphorylation of downstream survival effectors of GH/GHR signaling in HepG2-SR tumor xenografts. H&E staining demonstrates that PEGV-200 treatment was associated with expanded areas of hemorrhage and tumor lysis (black circles) in HepG2 (left panel) and HepG2-SR (right panel) tumor xenografts. Admixed within these areas were small nests of residual tumor cells (black arrows). Immunohistochemical staining shows expression of GHR in tumor xenografts, however, Ki-67+ cells decreased remarkably in tumors from mice treated with PEGV-200 vs. those from control mice and mice treated with SOR-5 alone. The arrows highlight the small foci of residual tumor cells. In addition, pAKT, pERK, and pIGF-IR proteins were almost entirely lacking within the expanded areas of tumor hemorrhage and lysis and were limited only to scattered clusters of residual viable tumor cells in mice treated with PEGV-200 (arrows). Conversely, control tumors and tumors treated with SOR-5 alone showed high levels of expression of the activated forms of these survival proteins. Original magnification is ×100 for H&E, Ki-67, and the SOR-5+PEGV-200 photomicrographs and ×400 for all other photomicrographs. [file Image_4.tif]
